# Supplementary material for: Identification of Freezing-Responsive microRNAs and Their Targets in Chinese Jujube by Small RNA and Degradome Sequencing
Source: Int J Mol Sci. 2026 Sep 4;27(17):7912. doi: 10.3390/ijms27177912 (PMC13566890; doi:10.3390/ijms27177912)
Supplement: Supplementary file 1 [file ijms-27-07912-s001.zip › Supplementary Material S2/novel_miR_106.pdf]

Provisional ID	:	novel_miR_106
Score total	:	0.4
Score for star read(s)	:	-1.3
Score for read counts	:	0
Score for mfe	:	0.1
Score for randfold	:	1.6
Score for cons. seed	:	
Total read count	:	159
Mature read count	:	159
Loop read count	:	0
Star read count	:	0

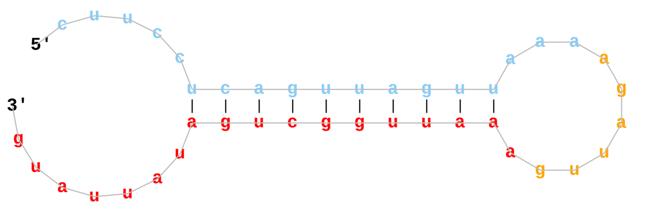

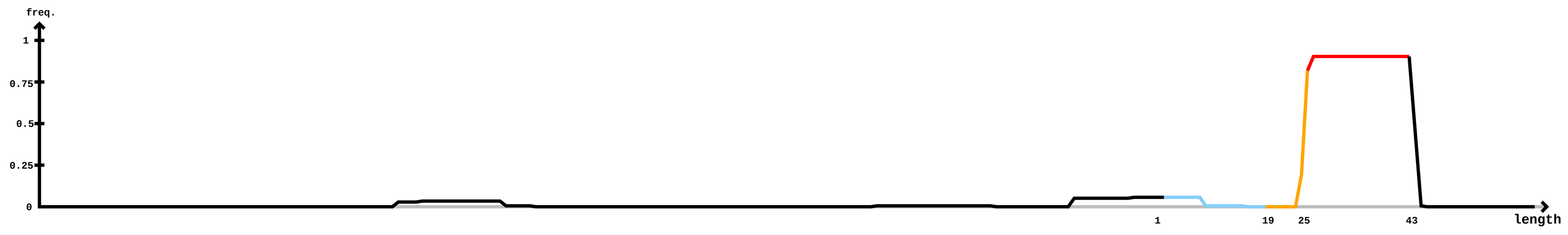

Star Mature

```
5'- ggaauuauuuuuaauaucaauuuauaaguaaaaaaacauucugcuacauuugaauuaccaaaacaagaacucuauguuagaguugaagccccaaaaugagaauugggaacuuuucugauaggggaucagggugguuuuccuuggagauggaaaauucacccaccauguuuuaucaauuugcaaauucuuccucaguuaguuaaaagauugaaauuggcugauauuaugaugauacuagguagcauagaua -3'
    ....((((((.(((.......))).))))))..........(((((.((((.....))))....(((((((...)))))))....((((...((.((((..(....)..)))).)).))))....(((((((((((........))))))).))))))).....((.((((((...............((((((((((.........))))))))))).....)))))).))...))))))......
```

exp read	reads	mm	sample
Aaaaacaagaacucuaug	5	1	LT-2
aGaagaacucuauguuaga	1	1	LT-1
ccuuggagauggaaaauuca	1	0	LT-3
aucaauuugcaaauucuuccuc	3	0	JCK-1
aucaauuugcaaauucuuccuc	2	0	JCK-2
aucaauuugcaaauucuuccuc	4	0	JCK-3
aaauucuuccucaguuagu	1	0	JCK-1
Aaaauuggcugauauuaug	1	1	LT-1
Aaaauuggcugauauuaug	1	1	LT-2
Aaaauuggcugauauuaug	12	1	LCK-3
Aaaauuggcugauauuauga	10	1	LCK-1
Aaaauuggcugauauuauga	1	1	LT-3
Aaaauuggcugauauuauga	9	1	LCK-2
aaauuggcugauauuauA	1	1	LT-1
aaauuggcugauauuaug	17	0	LT-3
aaauuggcugauauuaug	7	0	LCK-2
aaauuggcugauauuaug	2	0	JCK-3
aCauuggcugauauuaug	1	1	LT-3
Caauuggcugauauuaug	1	1	LCK-3
aaauuggcugauauuaug	11	0	LT-1
Caauuggcugauauuaug	1	1	LT-3
aaauuggcugauauuaug	8	0	LCK-3
aaauuggcugauauuaug	5	0	JCK-1
aaauuggcugauauuaug	6	0	LT-2
aaauuggcugauauuauA	13	1	LCK-1
aaauuggcugauauuaug	5	0	JCK-2
aaauuggcugauauuauA	1	1	LT-3
aaauuggcugauauuaug	2	0	LCK-1
aaauuggcugauauuauA	15	1	LCK-2
aaauuggcugauauuauga	1	0	JCK-2
aaauuggcugauauuauga	6	0	LCK-3
aaauuggcugauauuauga	2	0	JCK-3
aaauuggcugauauuauga	1	0	LT-1
aaauuggcugauauuauga	3	0	LT-3

```
                                                                                                                                                                                                                         Star              Mature
ggaauuauuuuuaauaucaauuuauaaguaaaaaaacauucugcuacauuugaauuaccaaaacaagaacucuauguuagaguugaagccccaaaaugagaauugggaacuuuucugauaggggaucagggugguuuuccuuggagauggaaaauucacccaccauguuuuaucaauuugcaaauucuuccucaguuaguuaaaaagauugaaauuggcugauauuaugaugauacuagguagcauagaua
...................................................................................................................................................................................................................aaauuggcugauauuaugau....................	1	0	LT-1
....................................................................................................................................................................................................................aauuggcugauauuauga.....................	1	0	LT-1
....................................................................................................................................................................................................................aCuuggcugauauuauga.....................	1	1	LT-2
....................................................................................................................................................................................................................aauuggcugauauuauga.....................	8	0	LT-2
....................................................................................................................................................................................................................aauuggcugauauuauga.....................	5	0	LT-3
```
